# Prevalence and factors associated with poor mental health among healthcare professionals in low- and lower-middle-income countries: a systematic review protocol

**DOI:** 10.1186/s13643-019-1201-7

**Published:** 2019-11-29

**Authors:** Julia Lohmann, Denny John, Aso Dzay

**Affiliations:** 1 Department of Global Health and Development, London School of Hygiene & Tropical Medicine, London, UK; 2 Heidelberg Institute of Global Health, University Hospital Heidelberg, Heidelberg, Germany; 3 Campbell South Asia, New Delhi, India; 4 ICMR-National Institute of Medical Statistics, New Delhi, India

**Keywords:** Systematic review, Mental health, Wellbeing, Health workers, LLMIC

## Abstract

**Background:**

A healthy and productive health workforce is central to a well-functioning health system. However, health workers are at high risk of poor psychological wellbeing due to their particularly strenuous work demands. While mental health of health workers is a well-researched issue in high-income countries, research from low- and lower-middle-income countries (LLMIC) has begun to emerge only recently. The review aims to synthesize this body of research, specifically to assess the prevalence of mental health issues among health workers in LLMIC, to identify factors associated with good or poor mental health, and to highlight gaps in knowledge.

**Methods:**

We will perform a systematic search of the published English and French language literature (from inception onwards) in MEDLINE, EMBASE, and PsycINFO. Eligible for inclusion are observational studies (e.g., cross-sectional, case-control, or cohort) and control arms of randomized controlled trials reporting investigations on the nature, prevalence, and factors associated with mental health or psychological wellbeing among formally trained health professionals and health associate professionals delivering health services in formal healthcare facilities in LLMIC. The primary outcomes will be burnout, depression, and general psychological wellbeing. Secondary outcomes include other specific mental health diagnoses, as well as general psychological stress, distress and/or trauma if work-related and explicitly framed as a mental health issue. Two authors will independently examine the studies against the eligibility criteria in the stages of title, abstract, and full-text study selection, as well as assess the risk of bias in included studies using standard checklists depending on study design. Disagreements will be resolved in discussion with the third author. Data will be extracted from included studies using a predefined and piloted coding framework. Given the anticipated heterogeneity of studies, we do not expect to be able to conduct meta-analysis and plan to summarize the extracted data in narrative form. The framework method will be used to organize narrative data by subthemes and explore patterns.

**Discussion:**

In assessing the prevalence of mental health issues among healthcare professionals in LLMIC and identifying factors associated with positive or poor mental health, the review aims to synthesize all possible available information for policy makers and health system managers on a potentially highly important but not yet much-discussed issue and to highlight gaps in currently available knowledge.

**Systematic review registration:**

International Prospective Register of Systematic Reviews PROSPERO (registration number CRD42019140036)

## Introduction

A competent, responsive, and productive health workforce is one of the six essential building blocks of a well-performing health system capable of providing universal access to high-quality care [1, 2]. To sustain their availability and productivity over time, ensuring health workers’ physical and mental health is critical, especially considering that they have been identified as being at high risk of poor psychological well-being due to their particular work demands [3, 4].

In high-income countries (HIC) and at an international level, the importance of keeping the workforce healthy and the key role of enabling and supportive working conditions have long been recognized and well researched. For instance, a 2006/2007 survey of over 60,000 nurses in twelve European countries and the USA found burnout rates ranging from around 10% in the Netherlands up to 78% in Greece [5]. Various factors associated with poor psychological wellbeing have been identified, such as excessive workload, inter- and intra-professional conflict, adverse management styles and poor management support, lack of autonomy, shift work, and effort-reward imbalance. Poor mental health has been linked to low quality of care [5], patient safety issues [6], poor empathic ability [7], and absenteeism [8].

In low- and lower-middle-income countries (LLMIC), occupational health and particularly psychological wellbeing are not part of the mainstream human resources for health (HRH) applied discourse and academic literature yet. However, a number of quantitative studies reporting on mental health or psychological wellbeing of health workers in LLMIC have recently been published and point at mental health issues in the health workforce being a substantial concern. This notion was supported by a recent systematic review of this emerging body of research which included studies published until 2016 [9]. However, the review focused only on burnout, among only primary care providers. This review did not assess the quality of included studies, included only English language studies, and further appears to be not quite inclusive of all relevant available studies.

We therefore intend to update and expand from the previously published review. Most importantly, we will broaden the definition of mental health to include syndromes other than burnout in alignment with the World Health Organization’s understanding of mental health as “a state of well-being in which every individual realizes his or her own potential, can cope with the normal stresses of life, can work productively and fruitfully, and is able to make a contribution to her or his community” [10]. Specifically, we conceptualize mental health as a spectrum from perfect wellbeing at one end to clinically relevant, severe mental illness incapacitating a person’s daily functioning at the other end. In line with the most commonly used taxonomy of determinants and consequences of mental illness [11] and in a work context, we further conceptualize psychological wellbeing as embedded within a complex system of determinants and consequences at the individual, organizational, and broader systemic levels. In alignment with recent developments in country classification [12, 13] and to reflect where HRH and other health system challenges tend to be most severe [14, 15], we will further include studies from low-income countries as well as lower-middle-income countries, rather than all middle-income countries as in Dugani et al. [9]. We will include health workers at all levels of care but limit our review to formally trained health professionals and health associate professionals. We will purposely exclude community and other lay and traditional health workers as well as technician, auxiliary, management, and other non-patient-facing personnel, as these staffing categories and their exact roles within health systems differ widely between countries and are therefore difficult to compare. Finally, we will include both English and French language articles.

In assessing the prevalence of mental health issues among healthcare professionals in LLMIC and identifying factors associated with positive or poor mental health, the review aims to synthesize all possible available information for policy makers and health system managers on a potentially highly important but not yet much-discussed issue, and to highlight gaps in currently available knowledge.

## Methods

### Protocol and registration

The present review protocol is being reported in accordance with the Preferred Reporting Items for Systematic Reviews and Meta-Analyses Protocols (PRISMA-P) statement [16] (see PRISMA-P checklist in Additional file 1). This review protocol was registered within the International Prospective Register of Systematic Reviews (PROSPERO) (registration number: CRD42019140036). Any important protocol amendments will also be documented in PROSPERO.

### Eligibility criteria

#### Participants

The review will consider studies that include

- Formally trained health professionals and health associate professionals, as defined in the ILO international classification of occupations used by the World Health Organization [17, 18], specifically medical doctors (category 221), nursing and midwifery professionals (222), and nursing and midwifery associate professionals (322)
- Working in formal healthcare facilities (public, private not-for-profit, private for-profit)
- Working in low- and lower-middle-income countries (LLMIC) as per the World Bank’s classification [12] (see Additional file 2)

We will not consider studies focusing exclusively on non-clinical or not formally trained personnel, such as health facility staff in a purely managerial or administrative function, traditional or untrained lay health workers, cleaners, etc.; studies conducted in community settings, studies focusing on individuals working exclusively outside formal health facilities; studies with an exclusive focus on upper-middle- or high-income countries; and studies on LLMIC migrant health professionals working in high-income countries.

#### Outcomes

The primary outcomes will be

- Burnout, measured with both well-established (e.g., Maslach Burnout Inventory (MBI) [19]) and less well-established or self-developed measures
- Depression, measured with both well-established (e.g., Beck Depression Inventory [20]) and less well-established or self-developed measures
- General psychological wellbeing, measured with both well-established (e.g., WHO-5 Wellbeing Index [21], Warwick Edinburg Mental Wellbeing Scale [22], Mbindyo’s measure [23]) and less well-established or self-developed measures

Secondary outcomes include other specific mental health/distress diagnoses or concepts (as detected by generic search terms for mental health/distress and/or summarized under respective MeSH terms) as well as general psychological stress and distress if work-related and explicitly framed as a mental health issue distress, and/or trauma. We will not consider studies on stress and job or life satisfaction without specific reference to mental health.

#### Types of studies

We will include studies reporting investigations of the nature, prevalence, and determinants of mental health or psychological wellbeing issues among health workers in low- and lower-middle-income countries. Specifically, relevant study designs include observational studies (cross-sectional, cohort, or case-control studies) and control arms of randomized controlled trials published in English and French language. If any study compared the prevalence in a country from LLMIC with a high-income country, information only from the LLMIC will be included. Where multiple papers were generated from the same data set with the same outcome, only the most relevant/recent paper will be included. However, if multiple papers were generated from the same data set with different outcomes or on different subpopulations, all papers will be included.

We will not consider qualitative studies. We will also not consider previously published systematic reviews, opinion pieces, commentaries, and policy briefs. Further, we will not include conference abstracts unless a full text can be found.

### Information sources

We will search for eligible studies published from inception onwards, in English or French language, in MEDLINE, EMBASE, and PsycINFO.

### Search

A comprehensive sensitive search strategy will be employed using key words in alignment with the above inclusion and exclusion criteria. A database record will be maintained at each stage of the review process detailing how the search was undertaken including the results of the search strategy.

The search strategy will include a combination of subject terms and free-text terms and will be combined with “OR” and “AND” operators. The search terms will include terms from three categories in alignment with the inclusion and exclusion criteria: (1) geographic focus: list of all LLMIC as well as generic terms for the geographic scope; (2) outcomes: specific terms for burnout, depression, and psychological wellbeing, generic terms for mental health/illness and work-related psychological stress/distress/trauma, and terms for specific common measurement instruments (e.g., MBI); and (3) population: generic terms for healthcare professionals as well as terms for specific health worker cadres. MeSH terms will be explored and included where available. The search strategy for MEDLINE is given in Additional file 3.

Results of the search strategy will be stored and managed in Mendeley, including removal of duplicates. EPPI-Reviewer 4 will be used to support and document the study selection, data extraction, and data synthesis.

### Study selection

Two authors will independently examine the studies against pretested eligibility criteria in the stages of title, abstract, and full-text study selection. None of the screening authors will be blind to any details of the studies. In the title and abstract screening stage, two authors (JL and AD) will independently screen initial subsets of studies until convergence is reached. Discrepancies will be discussed with the third author (DJ) and criteria refined in the process if necessary. The title and abstract screening process will then be finalized in single screening. Subsequently, full-text records of the selected abstracts will be retrieved. Full-text screening will be performed in full independent double screening (JL and AD). Full-text records selected for inclusion by both the authors will be included in the review. Any disagreements during this stage will be resolved through discussion and consensus with the third author (DJ). We will contact study authors, where appropriate, to clarify missing or inadequate information to determine study eligibility. The primary reason for the exclusion of articles will be documented at the full-text stage of study selection. A final list of articles will be prepared for data extraction.

### Data collection process

Data will be extracted from papers using a coding framework which will be finalized and piloted once initial screening has been performed and the research team has a better overview of the exact themes covered by existing studies. Data will be extracted by two independent researchers (JL and AD) until a high level of agreement is reached. Data extraction will then be finalized by one of the authors. Authors of primary publications (e.g., corresponding authors) will be contacted by email for data clarifications or missing outcome data, as necessary.

### Data items

From each included study, the following information will be extracted:

- Study information
  - ◦ Year of study conduct
  - ◦ Setting (country, specific geographic location(s), urban/rural, level of care)
  - ◦ Study design (cross-sectional/case-control/cohort/RCT control arm)
  - ◦ Sampling strategy (random/convenience/other)
  - ◦ Study funding source
- Study participants
  - ◦ Health worker cadre(s) (as per respective local definition, to be combined in the analytical stage to the extent possible and appropriate)
  - ◦ Sample size (per study subgroups if available)
  - ◦ Key demographic characteristics (age, sex, seniority in healthcare)
- Outcome(s)
  - ◦ Outcome measured (burnout, depression, psychological wellbeing; other psychological and/or occupational stress/distress/trauma)
  - ◦ Outcome measurement instrument used (incl. information on whether and how validated)
  - ◦ Type of measurement (percentage/odds ratio/risk ratio/measurement on psychometric scale)
- Factors associated with mental health
  - ◦ Factor(s) measured
  - ◦ Measurement instruments used (incl. information on whether and how validated)
- Study findings related to prevalence outcomes (per study subsample if available)
  - ◦ Result
  - ◦ Response rate
  - ◦ Any other results
  - ◦ Any additional relevant methodological information (unit of analysis if not individual, statistical methods used, weighing, etc.)
- Study findings related to factors associated with mental health (per factor and study subsample if available)
  - ◦ Result
  - ◦ Response rate
  - ◦ Any other results
  - ◦ Any additional relevant methodological information (unit of analysis if not individual, statistical methods used, weighing, etc.)
- Reported strengths and limitations as well as strategies to overcome limitations
- Key conclusions by the authors

### Risk of bias in individual studies

Two authors will independently assess the risk of bias at outcome level for all included studies by assessing their methodological quality using the JBI critical appraisal checklists for studies reporting prevalence data [24], for analytical cross-sectional studies [25], and for any other study design should this be relevant. Studies reporting both prevalence and factors associated with mental health will be assessed with both checklists in line with the analytical strategy detailed below. Any disagreements will be resolved through discussion and consensus with the third author.

### Data synthesis

Given our current knowledge of the literature, we anticipate to find relatively few comparable studies due to the large heterogeneity in the mental health measures used, their underlying mental health and psychological wellbeing conceptualizations, and the samples in which mental health was measured. Similarly, we anticipate high heterogeneity in factors associated with mental health, both conceptually and in regard to measurement. We consequently do not anticipate being able to conduct meaningful meta-analysis, neither regarding prevalence nor factor associated with mental health. We therefore plan a narrative synthesis of the findings of the included studies, following the Cochrane recommendations [26]. We plan to analyze data on prevalence separate from such on factors associated with mental health. For both parts, we will employ the framework method [27] to organize data by the subthemes and explore patterns. Subthemes will include the themes outlined in the data item section above, as well as study quality (risk of bias assessment) as evaluated as part of the review. We will present results by subtheme both in narrative form as well as using tables and visuals to facilitate reading to the extent possible.

Although we do not expect so, there is a chance that we might find a sufficient number (which we define as 5 or more) of studies with meaningfully comparable samples having measured burnout with the Maslach Burnout Inventory. In this case, we will estimate the pooled MBI-measured burnout prevalence from the reported prevalence using R software. Forest plots will be generated displaying prevalence with the corresponding 95% confidence interval (CI; asymptotic Wald) for each study using a random-effects model. Should sufficient studies be available, meta-regression will be conducted to identify sources of between-study heterogeneity in the pooled prevalence estimates. To examine the magnitude of the variation between the studies, heterogeneity will be identified by using the I^2^ measure and its CI. The degree of heterogeneity will be assessed into low, moderate, and high using cutoffs as prescribed in the Cochrane Handbook [26]. Statistical significance will be determined by the *χ*^2^ statistic for Q, with a *p* value < 0.05 indicating significance. Assessment of reporting biases will be conducted using funnel plots and Egger’s method to assess asymmetry if more than 10 studies are included in the meta-analysis. To the extent possible, stratified prevalence will be generated by key sample characteristics (e.g., health worker cadre, level of care). Sensitivity analysis will be conducted to verify the robustness of the study conclusions, assessing the impact of methodological quality, study design, sample size, and the effect of missing data as well as the analysis methods on the review results. Sensitivity analysis will also be conducted to investigate suspected funnel plot asymmetry due to publication bias if any. Should any meta-analysis be possible, we will assess the strength of the body of evidence using the GRADE guidelines [28]. If sufficient data is not available, studies with missing data will be omitted from a potential quantitative data synthesis. We do not plan to conduct any meta-analysis for prevalence estimates derived with measurement tools used in less than 5 studies, or for findings regarding factors associated with mental health due to the above-described high anticipated level of heterogeneity.

### Patient and public involvement

DJ is a trained physical therapist and has experience of working in tertiary care hospitals and community-based rehabilitation programs in India. AD is a medical doctor with work experience at all levels of care in Iraq. Beyond this, we did not involve any patients or the public in our review.

## Discussion

This systematic review (and meta-analysis, if any) will provide an overview of the prevalence of poor mental health among healthcare providers in LLMICs, as well as of factors associated with good or poor mental health.

The review will be published in a peer-reviewed journal and presented at appropriate conferences. The manuscript will be structured using the meta-analysis of observational studies in epidemiology (MOOSE) guidelines [29]. Results will further be disseminated to the applied HRH community through channels such as those of the Global Alliance for Nursing and Midwifery and the World Health Organization’s HRH Exchange.

Among the potential challenges we anticipate are most importantly at review level decisions as to whether studies assessing wellbeing or stress should be classified as dealing with mental health issues or not, as well as at study level by a high level of heterogeneity in study populations and measures and a dearth of validation studies of the used measurement tools, making generalized interpretation and cross-country comparison difficult. We will strive to mitigate these as well as further anticipated and unanticipated challenges by highlighting decision criteria, limitations, gaps in knowledge, and resulting threats to interpretation in a highly transparent manner, and by general caution in aggregation and comparison.

Despite these potential limitations, we are convinced that review will make an important contribution to the HRH applied and academic discourse by providing a summary of current evidence on a potentially highly important but not yet much-discussed issue, and to highlight gaps in currently available knowledge.

## Supplementary information

**Additional file 1.** PRISMA-P checklist.

**Additional file 2.** List of low- and lower-middle income countries (LLMIC) as per the World Bank’s classification in the 2019 fiscal year.

**Additional file 3.** Search strategy for MEDLINE.

## Data Availability

Not applicable

## Abbreviations

CI: Confidence interval
HIC: High-income country
HRH: Human resources for health
ILO: International Labor Organization
LLMIC: Low- and lower-middle-income country
MBI: Maslach Burnout Inventory
