# Supplementary material for: Prevalence and factors associated with poor mental health among healthcare professionals in low- and lower-middle-income countries: a systematic review protocol
Source: Syst Rev. 2019 Nov 29;8:294. doi: 10.1186/s13643-019-1201-7 (PMC6884904; doi:10.1186/s13643-019-1201-7)
Supplement: Supplementary file 2 — Additional file 2. List of low- and lower-middle income countries (LLMIC) as per the World Bank’s classification in the 2019 fiscal year. [file 13643_2019_1201_MOESM2_ESM.docx]

**List of low- and lower-middle income countries (LLMIC) as per the World Bank’s classification in the 2019 fiscal year [12]**

LOW-INCOME ECONOMIES ($1,025 OR LESS)

| Afghanistan | Guinea-Bissau |  | Sierra Leone |
| --- | --- | --- | --- |
| Benin | Haiti |  | Somalia |
| Burkina Faso | Korea, Dem. People's Rep. |  | South Sudan |
| Burundi | Liberia |  | Syrian Arab Republic |
| Central African Republic | Madagascar |  | Tajikistan |
| Chad | Malawi |  | Tanzania |
| Congo, Dem. Rep | Mali |  | Togo |
| Comoros | Mozambique |  | Uganda |
| Eritrea | Nepal |  | Yemen, Rep. |
| Ethiopia | Niger |  | Zimbabwe |
| Gambia, The | Rwanda |  |  |
| Guinea | Senegal |  |  |

LOWER-MIDDLE INCOME ECONOMIES ($1,026 TO $3,995)

| Angola | Indonesia | Philippines |
| --- | --- | --- |
| Bangladesh | Kenya | São Tomé and Principe |
| Bhutan | Kiribati | Solomon Islands |
| Bolivia | Kyrgyz Republic | Sudan |
| Cabo Verde | Lao PDR | Timor-Leste |
| Cambodia | Lesotho | Tunisia |
| Cameroon | Mauritania | Ukraine |
| Congo, Rep. | Micronesia, Fed. Sts. | Uzbekistan |
| Côte d'Ivoire | Moldova | Vanuatu |
| Djibouti | Mongolia | Vietnam |
| Egypt, Arab Rep. | Morocco | West Bank and Gaza |
| El Salvador | Myanmar | Zambia |
| Eswatini | Nicaragua |  |
| Ghana | Nigeria |  |
| Honduras | Pakistan |  |
| India | Papua New Guinea |  |
