# Supplementary material for: Prevalence and factors associated with poor mental health among healthcare professionals in low- and lower-middle-income countries: a systematic review protocol
Source: Syst Rev. 2019 Nov 29;8:294. doi: 10.1186/s13643-019-1201-7 (PMC6884904; doi:10.1186/s13643-019-1201-7)
Supplement: Supplementary file 3 — Additional file 3. Search strategy for MEDLINE. [file 13643_2019_1201_MOESM3_ESM.pdf]

Database: Ovid MEDLINE(R) and Epub Ahead of Print, In-Process & Other Non-Indexed Citations, Daily and Versions(R) <1946 to July 12, 2019>
Search Strategy:

----

1 exp Mental Health/ (34360)
2 (mental adj3 health).mp. (178833)
3 (mental adj3 hygiene).mp. (3215)
4 exp Burnout, Psychological/ (10720)
5 (psychological adj3 burnout).mp. (435)
6 (professional adj3 burnout).mp. (10828)
7 burnout.mp. (14759)
8 Motivation/ or motivation*.mp. (120884)
9 exp Occupational Stress/ (11547)
10 (occupational adj3 stress).mp. (3201)
11 (work adj3 stress).mp. (5484)
12 exp Stress, Psychological/ (123496)
13 (psychological adj3 stress).mp. (118717)
14 exp Mental Fatigue/ (1783)
15 (mental adj3 fatigue).mp. (2575)
16 (compassion adj3 fatigue).mp. (750)
17 (alert adj3 fatigue).mp. (231)
18 (mental adj3 distress).mp. (2186)
19 psychological well-being.mp. (8165)
20 (mental adj3 wellbeing).mp. (1207)
21 (emotional adj3 exhaustion).mp. (2317)
22 exp Depersonalization/ (1498)
23 depersonali#ation.mp. (3211)
24 (personal adj3 accomplishment*).mp. (1074)
25 exp Job Satisfaction/ (23807)
26 (job adj3 satisfaction).mp. (26789)
27 exp Work-Life Balance/ (384)
28 work-life balance.mp. (1347)
29 WHO-5 well-being index.mp. (92)
30 WHO5 wellbeing index.mp. (1)
31 maslach burnout inventory.mp. (1755)
32 warwick edinburg mental well-being scale.mp. (2)
33 mental hygiene.mp. (3172)
34 occupational burnout.mp. (142)
35 psychological distress*.mp. (17405)
36 exp Psychological Trauma/ or psychological trauma*.mp. (2274)
37 emotional exhaustion.mp. (2251)
38 personal accomplishment*.mp. (1041)
39 nurse burnout.mp. (117)
40 1 or 2 or 3 or 4 or 5 or 6 or 7 or 8 or 9 or 10 or 11 or 12 or 13 or 14 or 15 or 16 or 17 or 18 or 19 or 20 or 21 or 22 or 23 or 24 or 25 or 26 or 27 or 28 or 29 or 30 or 31 or 32 or 33 or 34 or 35 or 36 or 37 or 38 or 39 (455382)
41 exp Health Personnel/ (485920)
42 (health adj3 personnel).mp. (168435)
43 exp Physicians/ (130221)
44 physician*.mp. (540462)
45 nurse*.mp. (343665)
46 exp Nurses/ (85201)
47 clinician*.mp. (212859)
48 medical assistant*.mp. (811)
49 clinical officer*.mp. (302)
50 emergency department staff.mp. (373)

51 exp Hospitalists/ (1846)
52 hospitalist*.mp. (2750)
53 exp Medical Staff/ (26793)
54 medical staff.mp. (35304)
55 hospital medical staff.mp. (536)
56 exp Nursing Staff/ (64329)
57 nursing cadre.mp. (8)
58 primary care physician*.mp. (18799)
59 health worker*.mp. (19104)
60 nursing staff.mp. (72287)
61 health professional*.mp. (50090)
62 healthcare provider*.mp. (18941)
63 healthcare professional*.mp. (19328)
64 healthcare staff.mp. (1426)
65 birth attendant*.mp. (2163)
66 exp Midwifery/ (18605)
67 midwi#e*.mp. (34337)
68 exp Pharmacists/ (15594)
69 pharmacist*.mp. (34229)
70 exp Dentists/ (18323)
71 dentist*.mp. (124619)
72 primary health care provider*.mp. (684)
73 healthcare worker*.mp. (8556)
74 exp Allied Health Personnel/ (48173)
75 allied health personnel*.mp. (11565)
76 physical therapist*.mp. or exp Physical Therapists/ (6789)
77 physiotherapist*.mp. (7249)
78 community health officer*.mp. (50)
79 community health extension worker*.mp. (49)
80 junior community health extension worker*.mp. (2)
81 outpatient health care provider*.mp. (14)
82 outpatient healthcare provider*.mp. (8)
83 outpatient provider*.mp. (158)
84 exp Nursing Staff, Hospital/ (43830)
85 hospital nursing staff.mp. (137)
86 exp General Practitioners/ or general practitioner*.mp. (49931)
87 41 or 42 or 43 or 44 or 45 or 46 or 47 or 48 or 49 or 50 or 51 or 52 or 53 or 54 or 55 or 56 or 57 or 58 or 59 or 60 or 61 or 62 or 63 or 64 or 65 or 66 or 67 or 68 or 69 or 70 or 71 or 72 or 73 or 74 or 75 or 76 or 77 or 78 or 79 or 80 or 81 or 82 or 83 or 84 or 85 or 86 (1510223)
88 "Democratic People's Republic of Korea"/ (230)
89 (north korea or (democratic people* republic adj2 korea)).ti,ab. (378)
90 88 or 89 [LOW INCOME COUNTRIES IN EAST ASIA AND THE PACIFIC] (501)
91 Cambodia/ (3080)
92 cambodia.ti,ab. (3501)
93 Indonesia/ (9594)
94 indonesia.ti,ab. (10749)
95 Micronesia/ (1119)
96 Kiribati.ti,ab. (174)
97 Laos/ (1795)
98 (laos or (lao adj1 democratic republic)).ti,ab. (1766)
99 (marshall island* or caroline island* or ellice island* or gilbert island* or johnston island* or mariana island* or micronesia or pacific island*).ti,ab. (6261)
100 Mongolia/ (1668)
101 mongolia.ti,ab. (3595)
102 Myanmar/ (2204)
103 (myanmar or burma).ti,ab. (3621)

104 Papua New Guinea/ (3354)
105 Papua New Guinea.ti,ab. (4295)
106 Philippines/ (7960)
107 Philippines.ti,ab. (7612)
108 Timor-Leste/ (175)
109 Timor-Leste.ti,ab. (285)
110 Vanuatu/ (338)
111 Vanuatu.ti,ab. (593)
112 Vietnam/ (11436)
113 (Viet Nam or Vietnam).ti,ab. (13780)
114 or/91-113 [LOWER MIDDLE INCOME COUNTRIES IN EAST ASIA AND THE PACIFIC] (65618)
115 "Georgia (Republic)"/ (1726)
116 Kosovo/ (170)
117 Kosovo.ti,ab. (861)
118 Kyrgyzstan/ (1245)
119 (kyrgyzstan or kyrgyz republic or kirghizia or kirghiz).ti,ab. (909)
120 Moldova/ (668)
121 Moldova.ti,ab. (466)
122 Ukraine/ (15564)
123 Ukraine.ti,ab. (4272)
124 Uzbekistan/ (1881)
125 Uzbekistan.ti,ab. (1050)
126 or/115-125 [LOWER MIDDLE INCOME COUNTRIES IN EUROPE AND CENTRAL ASIA] (23980)
127 Haiti/ (2998)
128 Haiti.ti,ab. (2831)
129 127 or 128 [LOW INCOME COUNTRIES IN LATIN AMERICA AND THE CARIBBEAN] (3912)
130 Bolivia/ (2427)
131 Bolivia.ti,ab. (3007)
132 El Salvador/ (842)
133 El Salvador.ti,ab. (1177)
134 Honduras/ (1062)
135 Honduras.ti,ab. (1627)
136 Nicaragua/ (1420)
137 Nicaragua.ti,ab. (1744)
138 or/130-137 [LOWER MIDDLE INCOME COUNTRIES IN LATIN AMERICA AND CARIBBEAN] (8442)
139 Djibouti/ (215)
140 (Djibouti or French Somaliland).ti,ab. (356)
141 Egypt/ (13898)
142 Egypt.ti,ab. (12773)
143 Morocco/ (5376)
144 Morocco.ti,ab. (5028)
145 Tunisia/ (7768)
146 tunisia.mp. (9689)
147 Gaza.ti,ab. (950)
148 (Gaza or West Bank or Palestine).ti,ab. (2207)
149 or/139-148 [LOWER MIDDLE INCOME COUNTRIES IN MIDDLE EAST AND NORTH AFRICA] (38039)
150 Bangladesh/ (9999)
151 Bangladesh.ti,ab. (12033)
152 Bhutan/ (400)
153 Bhutan.ti,ab. (620)
154 exp India/ (97409)

155 India.ti,ab. (89511)
156 Pakistan/ (15843)
157 Pakistan.ti,ab. (15844)
158 Sri Lanka/ (5646)
159 Sri Lanka.ti,ab. (5822)
160 or/150-159 [LOWER MIDDLE INCOME COUNTRIES IN SOUTH ASIA] (176147)
161 Afghanistan/ (3019)
162 Afghanistan.ti,ab. (5441)
163 Nepal/ (7396)
164 Nepal.ti,ab. (8553)
165 or/161-164 [LOW INCOME COUNTRIES IN SOUTH ASIA] (16702)
166 Benin/ (1457)
167 (Benin or Dahomey).ti,ab. (3148)
168 Burkina Faso/ (3022)
169 (Burkina Faso or Burkina Fasso or Upper Volta).ti,ab. (3867)
170 Burundi/ (624)
171 Burundi.ti,ab. (750)
172 Central African Republic/ (755)
173 (Central African Republic or Ubangi-Shari).ti,ab. (964)
174 Chad/ (688)
175 Chad.ti,ab. (1070)
176 Comoros/ (290)
177 (Comoros or Comoro Islands or Mayotte or Iles Comores).ti,ab. (542)
178 "Democratic Republic of the Congo"/ (3909)
179 ((democratic republic adj2 congo) or belgian congo or zaire).ti,ab. (3943)
180 Eritrea/ (309)
181 Eritrea.ti,ab. (482)
182 Ethiopia/ (11219)
183 Ethiopia.ti,ab. (12906)
184 Gambia/ (2330)
185 Gambia.ti,ab. (2167)
186 Guinea/ (970)
187 (Guinea not (New Guinea or Guinea Pig* or Guinea Fowl)).ti,ab. (3779)
188 Guinea-Bissau/ (888)
189 (Guinea-Bissau or Portuguese Guinea).ti,ab. (977)
190 Liberia/ (1126)
191 Liberia.ti,ab. (1424)
192 Madagascar/ (3237)
193 (Madagascar or Malagasy Republic).ti,ab. (4410)
194 Malawi/ (4796)
195 (Malawi or Nyasaland).ti,ab. (6252)
196 Mali/ (2241)
197 Mali.ti,ab. (3260)
198 Mozambique/ (2189)
199 (Mozambique or Mocambique or Portuguese East Africa).ti,ab. (3207)
200 Niger/ (1130)
201 (Niger not (Aspergillus or Peptococcus or Schizothorax or Cruciferae or Gobius or Lasius or Agelastes or Melanosuchus or radish or Parastromateus or Orius or Apergillus or Parastromateus or Stomoxys)).ti,ab. (3172)
202 Rwanda/ (2201)
203 (Rwanda or Ruanda).ti,ab. (2690)
204 Senegal/ (5497)
205 senegal.ti,ab. (5317)
206 Sierra Leone/ (1398)
207 Sierra Leone.mp. (2300)
208 Somalia/ (1493)

209 Somalia.ti,ab. (1248)
210 South Sudan/ (121)
211 south sudan.ti,ab. (454)
212 Tanzania/ (10516)
213 (Tanzania or Tanganyika or Zanzibar).ti,ab. (12277)
214 Togo/ (1085)
215 (Togo or Togolese Republic).ti,ab. (1366)
216 Uganda/ (11106)
217 Uganda.ti,ab. (12802)
218 Zimbabwe/ (5550)
219 (Zimbabwe or Rhodesia).ti,ab. (5676)
220 or/166-219 [LOW INCOME COUNTRIES IN SUB-SAHARAN AFRICA] (105853)
221 Angola/ (940)
222 angola.ti,ab. (1292)
223 Cameroon/ (5116)
224 Cameroon.ti,ab. (6217)
225 Cape Verde/ (179)
226 (Cape Verde or Cabo Verde).ti,ab. (552)
227 Congo/ (1743)
228 (congo not ((democratic republic adj3 congo) or congo red or crimean-congo)).ti,ab. (2386)
229 Cote d'Ivoire/ (2989)
230 (Cote d'Ivoire or Ivory Coast).ti,ab. (3563)
231 Ghana/ (7344)
232 (Ghana or Gold Coast).ti,ab. (9370)
233 Kenya/ (14851)
234 kenya.mp. (20275)
235 Lesotho/ (397)
236 (Lesotho or Basutoland).ti,ab. (644)
237 Mauritania/ (415)
238 Mauritania.ti,ab. (567)
239 Nigeria/ (27016)
240 Nigeria.ti,ab. (26040)
241 Atlantic Islands/ (731)
242 (sao tome adj2 principe).ti,ab. (137)
243 Sudan/ (4532)
244 (Sudan not south sudan).ti,ab. (6938)
245 Swaziland/ (524)
246 Swaziland.ti,ab. (799)
247 Zambia/ (4210)
248 (Zambia or Northern Rhodesia).ti,ab. (4786)
249 or/221-248 [LOWER MIDDLE INCOME COUNTRIES IN SUB-SAHARAN AFRICA] (95057)
250 Syria/ (1612)
251 (Syria or Syrian Arab Republic).ti,ab. (1762)
252 Yemen/ (1312)
253 Yemen.ti,ab. (1675)
254 or/250-253 [LOW INCOME COUNTRIES IN MIDDLE EAST AND NORTH AFRICA] (4561)
255 Tajikistan/ (714)
256 Tajikistan.ti,ab. (525)
257 or/255-256 [LOW INCOME COUNTRIES IN EUROPE AND CENTRAL ASIA] (969)
258 90 or 114 or 126 or 129 or 138 or 149 or 160 or 165 or 220 or 249 or 257 (506782)
259 40 and 87 and 258 (3652)

****
